# Realized Genome Sharing in Heritability Estimation Using Random Effects Models

**DOI:** 10.1534/g3.119.0005

**Published:** 2019-03-22

**Authors:** Bowen Wang, Elizabeth Thompson

**Affiliations:** * Department of Statistics, University of Washington, Seattle, Washington 98195-4322; † Adobe Inc., San Jose, California 95110-2704

**Keywords:** kinship, random effects, asymptotic bias, missing heritability, model mis-specification

## Abstract

For heritability estimation using a two-component random effects model, we provided formulas for the limiting distribution of the maximum likelihood estimate. These formulas are applicable even when the wrong measure of kinship is used to capture additive genetic correlation. When the model is correctly specified, we showed that the asymptotic sampling variance of heritability estimate is determined by both the study design and the extent of variation in the kinship measure that constitutes the additive genetic correlation matrix. When the correlation matrix is mis-specified, the extent of asymptotic bias depends additionally on how the fitted correlation matrix differs from the truth. In particular, we showed in a simulation study that estimating heritability using a population-based design and the classic GRM as the fitted correlation matrix can potentially contribute to the ”missing heritability” problem.

DNA inherited from the same ancestral copy by related individuals is said to be identical by descent (IBD). In the case of defined pedigrees, we measure IBD relative to pedigree founders. In quantitative genetics, correlation of trait values between relatives has often been modeled as a function of genome shared IBD. A useful parameter for pairwise IBD is the kinship coefficient, the probability that DNA randomly chosen from each individual at the same locus is IBD. Pedigree kinship (Ψ), the expectation of kinship coefficient over realizations of descent in the pedigree, is a deterministic function of the pedigree relationship. However, genome-wide realized kinship (Φ), the average of kinship coefficients over all loci in the genome varies widely around this expected value (Hill and Weir 2011). Advances in genetic marker data technology and statistical methodology have enabled us to estimate both local and genome-wide IBD sharing very accurately (*e.g.*, Thompson 2013; Wang *et al.* 2017, and references therein). It is important to understand how differences in modeling genetic correlation between individuals affect outcome of statistical analyses.

We investigate the problem of heritability estimation using a random effects model of two components, where the trait values are sums of normally distributed additive genetic random effects and unique environmental random effects. The correlation structure of the additive genetic random effects is twice the matrix of some kinship measure. This model was used by Thompson and Shaw (1990) to show how eigen-transformation can significantly improve efficiency of EM algorithm to find the maximum likelihood estimates (MLEs) of the variance parameters. More recently, Visscher and Goddard (2015) used the same model to study asymptotic sampling variance (ASV) of heritability estimates. Raffa and Thompson (2016) considered hypothesis testing and construction of confidence intervals for heritability. These authors focused on the use of pedigree kinship (Ψ) under the assumption of correct model specification.

On the applied side, it has become popular to estimate heritability from population samples, where the genetic correlation structure takes the form of the classic Genomic Relationship Matrix (GRM). A population-based design avoids confounding due to shared environmental effects in close relatives. The classic GRM is constructed from identity by state (IBS) matching at genotyped markers, and is often referred to as the relatedness matrix (*e.g.*, Yang *et al.* 2010). Wang *et al.* (2017) showed that the classic GRM (at the element level) is an unbiased (under mild assumptions), but not very accurate estimator of twice the genome-wide realized kinship, especially for remote relatives. It is of interest to know how choices of kinship measures (*e.g.*, realized or pedigree) impact outcome of heritability estimation.

In this paper we study the asymptotic distributions of heritability estimates under the random effects model, when the genetic correlation structure is potentially mis-specified. We investigate how impact of mis-specifying genetic correlation structure on heritability estimation varies with respect to study design and the extent of variation in realized kinship. Finally, we discuss the use of estimated kinship to capture genetic correlation in a population-based design, and how it can contribute to the “missing heritability problem” (Maher 2008; Manolio *et al.* 2009).

## Methods

### The polygenic model

In quantitative genetics, phenotype values are often modeled using a linear mixed model, where fixed effects may include age, sex, or principle components derived from the matrix of SNP genotypes to account for population sub-structure. In this paper we assume the fixed effects have been correctly adjusted for, so that

y=g+e, (1)

where y are trait values after adjustment of fixed effects, $g∼N\left(0,\sigma_{g}^{2}G\right)$ are additive genetic effects and $e∼N\left(0,\sigma_{e}^{2}I\right)$ are residuals. $\sigma_{g}^{2}$ and $\sigma_{e}^{2}$ are unknown variance parameters, and G is the appropriate genetic correlation matrix. Total phenotypic variance is $\sigma^{2}=\sigma_{g}^{2}+\sigma_{e}^{2}$. The goal is to estimate heritability, $h^{2}=\sigma_{g}^{2}/\sigma^{2}$. We can parametrize the trait distribution in terms of $\theta =\left(h^{2},\sigma^{2}\right)$ as

$$y∼N\left(0,\left[h^{2}G+\left(1-h^{2}\right)I\right]\sigma^{2}\right).$$ (2)

We assume throughout the paper that the model described in (1) and (2) is true, and that $h^{2}$ and $\sigma^{2}$ are both positive. However, the fitted correlation matrix $G_{f}$ may differ from the true correlation matrix $G_{t}$. To investigate the asymptotic distributions of the MLEs and the effect of pedigree structure on the MLEs, we assume there are *m* mutually independent pedigrees of the same structure with finite pedigree size *n*.

### Correct model specification

When $G_{f}=G_{t}$, it follows from likelihood theory that the MLEs are consistent. Let the eigen-decomposition of $G_{f}$ be $\mathrm{TD}T^{T}$, where T is the orthogonal matrix of eigenvectors and D is the diagonal matrix of eigenvalues of $G_{f}$. The transformed trait

$$y^{\text{*}}=T^{T}y∼N\left(0,\left[h^{2}D+\left(1-h^{2}\right)I\right]\sigma^{2}\right).$$ (3)

The covariance matrix $\text{Var}\left(y^{\text{*}}\right)$ is diagonal, so that the log-likelihood function without the constant term is

$$\ell \left(h^{2},\sigma^{2};y^{\text{*}}\right)=-\frac{1}{2}\left[N\;\text{ln}\left(\sigma^{2}\right)+\sum_{i=1}^{N}\text{ln}\left(h^{2}\lambda_{i}+1-h^{2}\right)+\frac{1}{\sigma^{2}}\sum_{i=1}^{N}\frac{y_{i}^{\text{*}2}}{h^{2}\lambda_{i}+1-h^{2}}\right],$$ (4)

where N=mn is the total sample size, $\lambda_{i}$ is the *i*th eigenvalue of $G_{f}$ (and $G_{t}$ in the case). ASV(${\hat{h}}^{2}$) is given by, among others, Visscher and Goddard (2015) as

$$\text{ASV}\left({\hat{h}}^{2}\right)=\frac{2}{\sum_{i=1}^{N}\frac{{\left(\lambda_{i}-1\right)}^{2}}{{\left(h^{2}\lambda_{i}+1-h^{2}\right)}^{2}}-\frac{1}{N}{\left[\sum_{i=1}^{N}\frac{\lambda_{i}-1}{h^{2}\lambda_{i}+1-h^{2}}\right]}^{2}}.$$ (5)

### Model mis-specification

When $G_{f}\neq G_{t}$, let the matrix of differences be $\Delta =G_{t}-G_{f}$. The same eigen-decomposition and transformation based on the fitted correlation matrix $G_{f}$ leads to the same log-likelihood function (4), but the true distribution of the transformed trait is now

$$y^{\text{*}}=T^{T}y∼N\left(0,\left[h_{0}^{2}D+\left(1-h_{0}^{2}\right)I\right]\sigma_{0}^{2}+h_{0}^{2}\sigma_{0}^{2}T^{T}\Delta T\right),$$ (6)

where $\theta_{0}=\left(h_{0}^{2},\sigma_{0}^{2}\right)$ are the true parameter values.

Let $P\left(\theta_{0}\right)$ denote the true trait distribution in (6). The model space for fitting with $G_{f}$ is denoted by Q={Q(θ),θ∈Θ=[0,1]×[0,∞)}. If we let m→∞, the MLEs from fitting the wrong model, $\hat{\theta }=\left({\hat{h}}^{2},{\hat{\sigma }}^{2}\right)$, will converge in probability to $\theta_{1}=\left(h_{1}^{2},\sigma_{1}^{2}\right)$ that minimizes the Kullback-Leibler divergence (Kullback and Leibler 1951) between $P\left(\theta_{0}\right)$ and Q(θ) over the parameter space Θ (*e.g.*, Severini 2000). In our case

$$\left(h_{1}^{2},\sigma_{1}^{2}\right)\approx \underset{\left(h^{2},\sigma^{2}\right)}{\arg\;\min}\frac{1}{2}\text{ln}\left(\sigma^{2}\right)+\frac{1}{2N}\sum_{i=1}^{N}\left[\text{ln}\left(h^{2}\lambda_{i}+1-h^{2}\right)+\frac{\left(h_{0}^{2}\lambda_{i}+1-h_{0}^{2}\right)\sigma_{0}^{2}+h_{0}^{2}\sigma_{0}^{2}t_{i}^{T}\Delta t_{i}}{\left(h^{2}\lambda_{i}+1-h^{2}\right)\sigma^{2}}\right],$$ (7)

where $t_{i}$ is the *i*th eigenvector of $G_{f}$. It follows from likelihood theory that

$$\sqrt{N}\left(\hat{\theta }-\theta_{1}\right)\to_{d}N\left(0,J^{-1}K{\left(J^{-1}\right)}^{T}\right),$$ (8)

with

$$J=\underset{m\to \infty }{\text{lim}}E_{P\left(\theta_{0}\right)}\left[\frac{\partial^{2}\ell \left(\theta ;y^{\text{*}}\right)/N}{\partial \theta^{2}}|{}_{\theta =\theta_{1}}\right],$$ (9)

$$K=\underset{m\to \infty }{\text{lim}}E_{P\left(\theta_{0}\right)}\left[\frac{\partial \ell \left(\theta ;y^{\text{*}}\right)/N}{\partial \theta }\times {\left(\frac{\partial \ell \left(\theta ;y^{\text{*}}\right)/N}{\partial \theta }\right)}^{T}|{}_{\theta =\theta_{1}}\right],$$ (10)

where $\ell \left(\theta ;y^{\text{*}}\right)$ is the log-likelihood over all data. For instance, the first element of J before taking limit can be shown to be

$$E_{P\left(h_{0}^{2},\sigma_{0}^{2}\right)}\left[\frac{\partial^{2}\ell \left(h^{2},\sigma^{2};y^{\text{*}}\right)/N}{\partial h^{4}}|{}_{h_{1}^{2},\sigma_{1}^{2}}\right]=\frac{1}{2N}\sum_{i=1}^{N}\left[\frac{{\left(\lambda_{i}-1\right)}^{2}}{{\left(h_{1}^{2}\lambda_{i}+1-h_{1}^{2}\right)}^{2}}-\frac{2{\left(\lambda_{i}-1\right)}^{2}\left[\left(h_{0}^{2}\lambda_{i}+1-h_{0}^{2}\right)\sigma_{0}^{2}+h_{0}^{2}\sigma_{0}^{2}t_{i}^{T}\Delta t_{i}\right]}{\sigma_{1}^{2}{\left(h_{1}^{2}\lambda_{i}+1-h_{1}^{2}\right)}^{3}}\right].$$ (11)

Given $h_{0}^{2}$, $\sigma_{0}^{2}$, $G_{t}$ and $G_{f}$ for any finite N=mn, (7) can be solved numerically for $\left(h_{1}^{2},\sigma_{1}^{2}\right)$. J and K in (9) and (10) can be computed subsequently to obtain the asymptotic variance covariance matrix of the MLEs.

For the special case where $G_{f}$ is twice the pedigree kinship matrix (2Ψ) and $G_{t}$ corresponds to realized kinship over the causal genome, elements of Δ represent (twice the) deviations of realized kinship from its pedigree expectation. Such deviations have expectation 0 when samples are not ascertained by trait or by IBD sharing. Since the pedigree kinship matrix is fixed given the pedigree structure, we have $2\Psi_{i}=\sum_{j=1}^{n}t_{j}\lambda_{j}t_{j}^{T}$ for all *i* (pedigree index). It follows from (6) that Δ enters (7), (9) and (10) only in the form of $t_{j}^{T}\left(\sum^{\text{​}}\Delta_{i}/m\right)t_{j}$, which converges to 0 as m→∞. This implies fitting model (1) with $G_{f}=2\Psi$, regardless what is the true causal genome from which realized kinship is measured and captured in $G_{t}$, produces consistent MLEs. In addition, the ASV of the MLEs will be the same as in the case $G_{f}=G_{t}=2\Psi$, which can be easily computed using (5).

### Method of analysis

**Figure 1:**
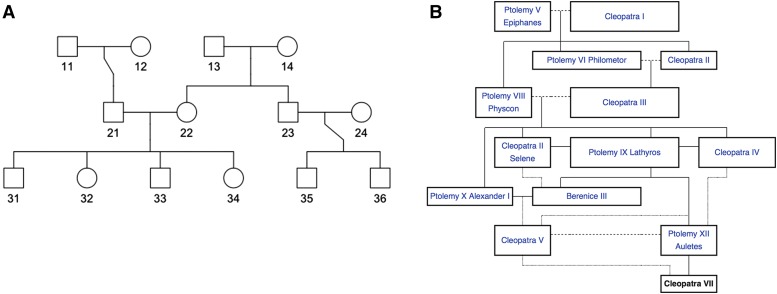
(A) Three-generation pedigree. (B) Cleopatra pedigree (Wikipedia 2018).

We conduct two separate simulation studies for this paper. The first simulation study serves to verify results presented in the previous sections, and to investigate the impact of study design and the extent of variation in kinship measure on heritability estimation. We consider four different types of designs: sibpairs, sibships of 14, three-generation pedigree of 14 members (Figure 1A), and the Cleopatra pedigree of 14 members (Figure 1B). Three types of kinship measures are used for $G_{t}$ and $G_{f}$: twice the pedigree kinship matrix (2Ψ), twice the genome-wide realized kinship matrix (2Φ), and twice the realized kinship matrix on chromosome 22 ($2\Phi^{\text{*}}$). These choices represent increasing amount of variation around the pedigree expectation.

We set total phenotypic variance $\sigma_{0}^{2}=1$ throughout, and use three heritability values, $h_{0}^{2}\in \left\{0.2,0.5,0.8\right\}$. In total, there are 108 simulation scenarios (4× designs, 9× $\left(G_{t},G_{f}\right)$ combinations, and 3× heritability values). The total sample size of the study is kept at N=1400 (*e.g.*, 700 sibpairs, or 100 sibships of 14). 500 simulation replicates are used to capture the empirical distributions of the MLEs in each of the 108 scenarios. Within each simulation replicate, we obtain $G_{t}$ and $G_{f}$ by simulating genome-wide descent in the pedigrees. Then we simulate trait data given $G_{t}$ and $h_{0}^{2}$ under model (1), and fit model (1) with $G_{f}$ to obtain the MLEs. Means and standard deviations of the empirical distribution of ${\hat{h}}^{2}$ are compared to those computed from (7) and (8) based on ($G_{t},G_{f}$).

The second simulation study investigates the potential impact of mis-specifying the genetic correlation structure in a population-based design. For ease of simulation, we use multiple independent pedigrees of second or third cousinships of various sizes. This block-diagonal structure might approximate that of a population sample that included clusters of more closely related individuals. The total sample size is kept at 2000 across different simulation conditions, so that in one of the conditions, for example, the sample consists of 200 independent third cousinships of 10. We consider three different kinship measures, each computed within cousinship. These are twice the genome-wide realized kinship matrix (2Φ), the classic GRM ($2{\hat{\Phi }}_{c}$), and the LD (linkage disequilibrium) weighted GRM ($2{\hat{\Phi }}_{w}$) introduced in Wang *et al.* (2017). Both the classic GRM and the LD weighted GRM are computed from simulated marker data. The LD weighted GRM represents a more accurate estimator of twice the realized kinship matrix than the classic GRM. For the two GRMs, we look at the cases both with and without constraining the diagonal terms (twice the self-kinship) to 1. True parameter values are set to $h_{0}^{2}=0.5$ and $\sigma_{0}^{2}=1$.

Simulations were performed using the R package *rres* (Wang 2018). Genome-wide realized kinship between each pair of individuals was measured as the proportion of shared genome (in genetic distances) over the 22 autosomes. To simulate marker genotypes, real haplotypes from the 1000 Genomes Project Phase 3 data (The 1000 Genomes Project Consortium 2015) were assigned to founders and populated down the pedigree given the simulated joint IBD pattern. A total of 169,751 markers were selected from all 22 autosomes based on minor allele frequency, marker spacing, and availability of genetic position. Details of the marker data can be found in Wang *et al.* (2017). These SNP markers were used to obtain the classic GRM and the LD weighted GRM using the *rres* package, with allele frequencies assumed known.

### Data availability

The 1000 Genomes Project Phase 3 data are available at http://www.1000genomes.org/data. Information on the set of 169,751 SNP markers used in the simulation studies can be found in the supplement of Wang *et al.* (2017).

## Results

In the first simulation study, there is generally very good agreement between the empirical and analytical results. Table 1 displays the results from the 9 combinations of ($G_{t},G_{f}$), for the sibship design and $h_{0}^{2}=0.5$. We see that ${\hat{h}}^{2}$ appears to be unbiased when $G_{f}=G_{t}$, and when $G_{f}=2\Psi \neq G_{t}$. In addition, $G_{f}=2\Psi$ resulted in very similar $\text{SE}\left({\hat{h}}^{2}\right)$ regardless of $G_{t}$, as predicted in the previous section. When $G_{f}\neq G_{t}$ and $G_{f}$ uses a more variable kinship measure than $G_{t}$ (in this case, realized kinship on a shorter causal genome), both the point estimates and sampling errors tend to be smaller than expected under correct model specification.

**Table 1:** Comparison of limits and asymptotic sampling standard errors of heritability estimates obtained by fitting model (1) and by using (**7**) and (**8**), with data on 100 independent sibships of 14. True parameters are $\left(h_{0}^{2},\sigma_{0}^{2}\right)=\left(0.5,1\right)$. Empirical distribution of ${\hat{h}}^{2}$ is constructed from 500 simulation replicates

$G_{t}$		2Ψ			2Φ			$2\Phi^{\text{*}}$		
$G_{f}$		2Ψ	2Φ	$2\Phi^{\text{*}}$	2Ψ	2Φ	$2\Phi^{\text{*}}$	2Ψ	2Φ	$2\Phi^{\text{*}}$
${\hat{h}}^{2}$	emp.	0.494	0.451	0.278	0.497	0.498	0.281	0.492	0.486	0.499
	analy.	0.500	0.453	0.279	0.500	0.500	0.282	0.498	0.490	0.500
$\text{SE}\left({\hat{h}}^{2}\right)$	emp.	0.063	0.052	0.032	0.065	0.060	0.032	0.064	0.059	0.032
	analy.	0.067	0.055	0.032	0.067	0.061	0.032	0.067	0.061	0.033

**Figure 2:**
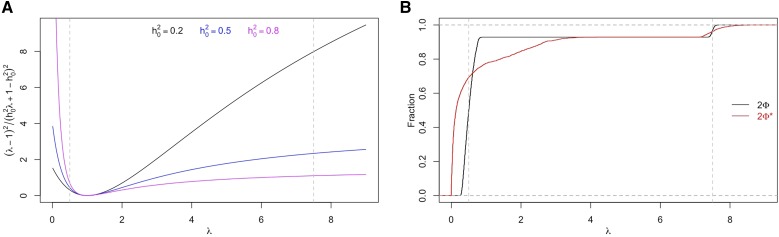
(A)${\left(\lambda -1\right)}^{2}/{\left(h_{0}^{2}\lambda +1-h_{0}^{2}\right)}^{2}$ evaluated at a range of λ values and $h_{0}\in \left\{0.2,0.5,0.8\right\}$. (B) Cumulative distributions of eigenvalues of 2Φ and $2\Phi^{\text{*}}$ from 100 simulated sibships of 14. Vertical dashed lines in both plots represent the two distinct eigenvalues of 2Ψ: 0.5 and 7.5.

When $G_{f}=G_{t}$, equation (5) suggests that pedigree structure affects ASV(${\hat{h}}^{2}$) only through eigenvalues of $G_{t}$. For large *N*, the first term in the denominator of (5),

$$\sum_{i=1}^{N}\frac{{\left(\lambda_{i}-1\right)}^{2}}{{\left(h_{0}^{2}\lambda_{i}+1-h_{0}^{2}\right)}^{2}},$$ (12)

dominates the second term. Since the summand in (12) is non-negative, eigenvalues that lead to bigger summand values have a higher tendency to reduce ASV(${\hat{h}}^{2}$). Figure 2A suggests that extreme eigenvalues have biggest impact in reducing sampling variance. Figure 2B shows that twice the realized kinship matrices on shorter genomic segments tend to produce more extreme eigenvalues. This provides intuition on why $\text{SE}\left({\hat{h}}^{2}\right)$ is much smaller when $G_{f}=G_{t}=2\Phi^{\text{*}}$, as compared to the other cases of correct model specification shown in Table 1.

**Figure 3:**
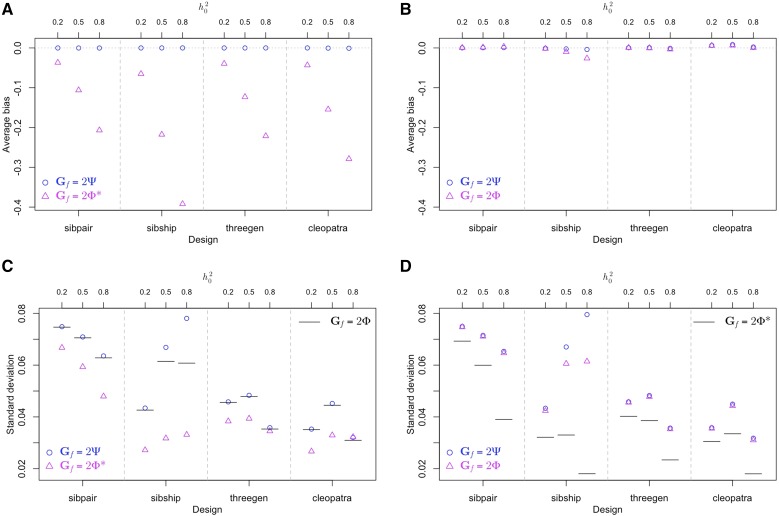
Simulation results for all four designs, three values of $h_{0}^{2}$ and various combinations of $\left(G_{t},G_{f}\right)$. (A) and (B) show the average bias of heritability estimates from 500 simulation replicates, when $G_{t}=2\Phi$ or $2\Phi^{\text{*}}$ respectively. (C) and (D) show the standard deviation of heritability estimates from 500 simulation replicates, when $G_{t}=2\Phi$ or $2\Phi^{\text{*}}$ respectively. In both (C) and (D), results obtained under correct model specification are shown in black bars as references.

Figure 3 shows simulation results from use of other designs and heritability values. We omit scenarios where $G_{t}=2\Psi$ since it cannot be true biologically. Most of the observations from earlier discussion of the sibship design also hold true for other designs. The one clear exception is that when $G_{t}=2\Phi$ and $G_{f}=2\Psi$, the impact of this specific model mis-specification on $\text{SE}\left({\hat{h}}^{2}\right)$ is very small when using other designs (compare blue circles to black bars in each scenario in Figure 3C). This is likely because the distribution of eigenvalues of the correlation matrix depends on the length of the causal genome as well as pedigree structure. For sibship design, a good proportion of eigenvalues associated with 2Φ are close to 0 (Figure 2B), which is not the case for other designs (results not shown). As a contrast, when $G_{t}=2\Phi^{\text{*}}$ and $G_{f}=2\Psi$, the impact of model mis-specification on $\text{SE}\left({\hat{h}}^{2}\right)$ is clear for all designs (compare blue circles to black bars in each scenario in Figure 3D). This is because the greater variation in $2\Phi^{\text{*}}$ due to shorter length of causal genome leads to the possibility of more extreme eigenvalues for each study design.

Lastly, we note that for any given combination of ($G_{t},G_{f}$), $\text{SE}\left({\hat{h}}^{2}\right)$ varies with $h_{0}^{2}$ quite differently across designs. For example, when $G_{f}=G_{t}=2\Phi$ the sibship design has slightly smaller $\text{SE}\left({\hat{h}}^{2}\right)$ than the three-generation design at $h_{0}^{2}=0.2$, but it is the opposite when $h_{0}^{2}=0.8$.

**Figure 4:**
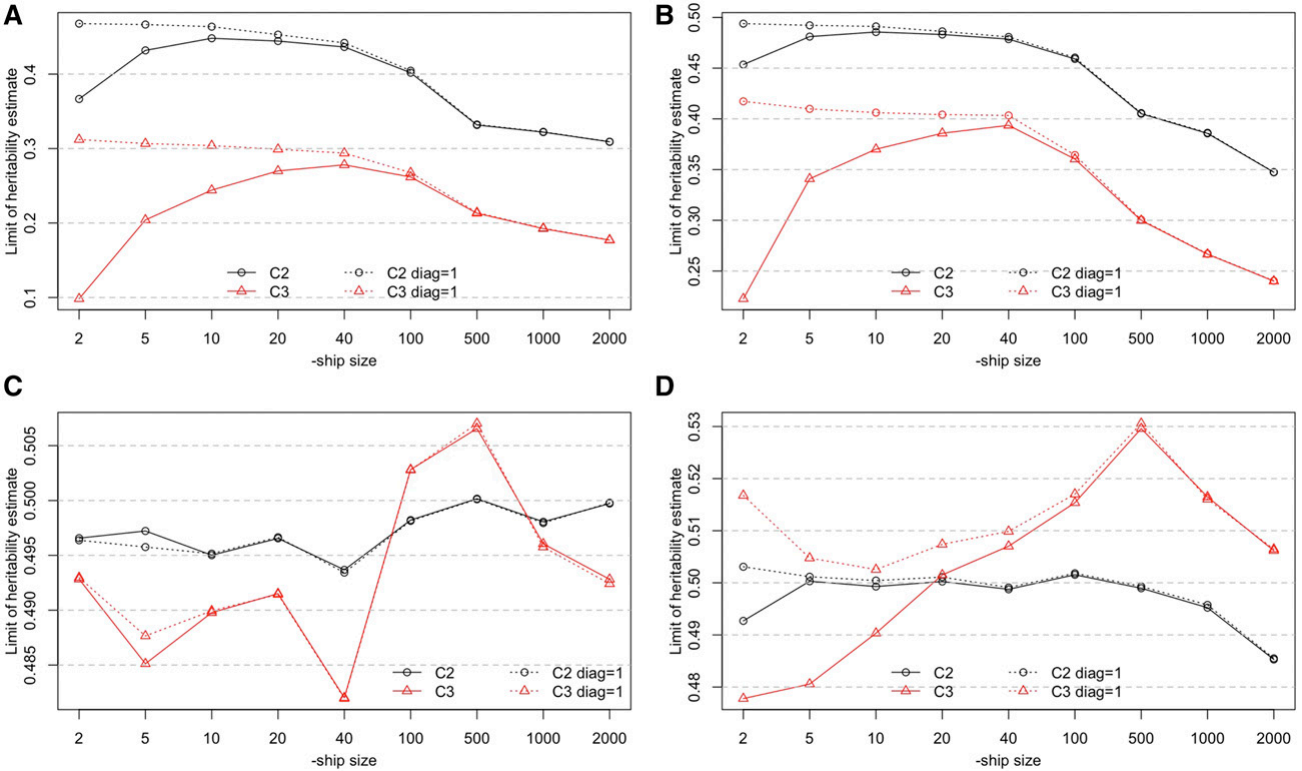
Limits of ${\hat{h}}^{2}$ when $h_{0}^{2}=0.5$ and $G_{f}\neq G_{t}$. Each sample contains multiple independent second (C2) or third (C3) cousinships of specific sizes. Three kinship measures considered are: twice the genome-wide realized kinship matrix (2Φ), the classic GRM ($2{\hat{\Phi }}_{c}$) and the LD weighted GRM ($2{\hat{\Phi }}_{w}$). In (A) and (B), $G_{t}=2\Phi$, $G_{f}=2{\hat{\Phi }}_{c}\text{ and }2{\hat{\Phi }}_{w}$ respectively. In (C) and (D), $G_{t}=2{\hat{\Phi }}_{c}$, $G_{f}=2\Phi \text{ and }2{\hat{\Phi }}_{w}$ respectively. The estimated matrices ($2{\hat{\Phi }}_{c}\text{ and }2{\hat{\Phi }}_{w}$) are used with and without constraining the diagonal terms to 1.

In the second simulation study, there is generally more bias in heritability estimates when the sample consists of third cousinships as opposed to second cousinships (Figure 4). This is in good agreement with the findings of Wang *et al.* (2017), where the authors showed it is relatively more difficult to estimate realized relatedness between more remote relatives. This observed association between level of bias and remoteness of relationship is further verified by additional simulations using full sibships, half sibships and first cousinships (results not shown).

Another observation from Figure 4 is that the decision to constrain the diagonal terms of $2{\hat{\Phi }}_{c}$ or $2{\hat{\Phi }}_{w}$ to 1 makes a big difference when pedigree sizes are small, but that difference erodes when pedigree sizes increase. A possible explanation is that apart from its effect on eigenvalues and eigenvectors in (7), $G_{f}\neq G_{t}$ induces bias in heritability estimates most directly from the matrix of difference, Δ. When we constrain the diagonal terms of $2{\hat{\Phi }}_{c}$ or $2{\hat{\Phi }}_{w}$ to 1, Δ will have 0’s on the diagonal. This is expected to have a bigger impact when pedigree sizes are small and the diagonal terms make up a bigger proportion of non-zero terms of Δ.

Figure 4A and 4B shows that when $G_{t}=2\Phi$, setting $G_{f}=2{\hat{\Phi }}_{w}$ produces less bias than $G_{f}=2{\hat{\Phi }}_{c}$ in all cases. This is expected since the LD weighted GRM is a more accurate estimator of twice the genome-wide realized kinship matrix. When constraining the diagonal terms of the estimated matrices to 1, bias increases with pedigree size for both second and third cousinships. Again, this is likely the result of the composition of non-zero terms in Δ: larger pedigree sizes imply higher proportion of non-zero terms. When not constraining the diagonal terms of the estimated matrices to 1, bias first decreases and then increases with pedigree size. A possible explanation is that in smaller cousinships, the diagonal terms of Δ take up a bigger proportion of all non-zero terms. While self-kinship can be estimated relatively accurately compare to realized kinship between remote cousins, the magnitude of deviations on the original scale, as captured in Δ, is greater for self-kinship (Wang *et al.* 2017). This means the diagonal terms of Δ could be more influential than off-diagonal terms on a per-entry basis, which lead to the bigger bias observed for smaller pedigrees.

Figure 4C and 4D show limits of heritability estimates when $G_{t}=2{\hat{\Phi }}_{c}$, and $G_{f}=2\Phi$ or $2{\hat{\Phi }}_{w}$. In both figures, the main observation is that the scale of bias is very small compare to that in Figure 4A and 4B. This matches our findings from the first simulation study: when $G_{t}$ and $G_{f}$ are associated with two kinship measures with zero expected differences (expectation over realization of descent on pedigree), we expect downward bias in heritability estimate if $G_{f}$ uses a more variable kinship measure than $G_{t}$, but not much bias the other way round (*e.g.*, Figure 3A and 3B). Wang *et al.* (2017) showed that both the classic GRM and the LD weighted GRM are unbiased estimators of twice the genome-wide realized kinship under certain assumptions, and the unbiasedness property held very well in simulation studies. Since the classic GRM is a less efficient estimator, it is a more variable measure of kinship compare to LD weighted GRM.

## Discussion

For heritability estimation using a two-component random effects model, we have provided formulas for the limits and the asymptotic sampling variances of the MLEs. These formulas are applicable even when the wrong kinship measure is used to capture additive genetic correlation. To study the impact of pedigree structure on asymptotic distribution of the MLEs, we have assumed that the sample consists of multiple independent pedigrees of the same structure. The assumption of a fixed pedigree structure is not required to derive equations (4) through (11) in **METHODS**, since log-likelihood sums over independent pedigrees of any structure. When different pedigree structures are present in the study sample, one only needs to replace the fixed pedigree size *n* with $n_{i}$ for pedigree *i* in those formulas and the asymptotic results are still valid.

Under correct specification of model (1), ASV of heritability estimates is a function of eigenvalues of the appropriate genetic correlation matrix, which in turn can depend on pedigree structure and variation in the corresponding kinship measure. For any pedigree structure and choice of kinship measure, the resulting kinship matrix can be easily obtained by simulation and subsequently used in the formulas we presented to assess effectiveness of the pedigree in heritability estimation under the assumed model. This is beneficial to pedigree selection in study design.

There can be bias in heritability estimate if the genetic correlation matrix has been mis-specified. The extent of the bias depends on both the true and the fitted correlation matrices, as well as the study design. When the true genetic correlation between individuals is captured by genome-wide realized kinship, one should use an accurate estimator of realized kinship to compute the fitted correlation matrix in order to reduce downward bias in heritability estimate. This is especially important when the study sample contains many remotely related individuals.

The popular classic GRM is not an accurate estimator of twice the realized kinship. When used in a population-based design, it can lead to substantial downward bias in heritability estimate if the truth ($G_{t}$) is that of twice the genome-wide realized kinship. The downward bias is expected to persist even if denser SNP panels are used to estimate realized kinship, as additional markers do not provide information without limit. In a follow up study of Wang *et al.* (2017), we found that increasing marker density by 4 times made little improvement in accuracy of the classic GRM estimator (results not shown). This choice of kinship estimator and study design can contribute to the “missing heritability” problem, among many other factors. On the other hand, using a more accurate kinship estimator to compute the fitted correlation matrix ($G_{f}$) is more robust to mis-specification of kinship measure.

When using a population-based design, it is common to remove an individual in each pair that has a kinship estimate exceeding certain threshold (*e.g.*, Yang *et al.* 2010). A threshold of 0.025 roughly corresponds to excluding closer relatives than second cousins (with pedigree kinship 0.0156). While more remote relatives often exist in a population sample, we only presented results on second and third cousinships in the second simulation study because they demonstrate all the important points from this simulation setup. The main reason for using a population-based design is not to eliminate IBD, but to avoid confounding due to shared environmental effects of closer relatives. Since our simulation models do not include shared environmental effects, our findings should generalize to studies that involve more remote relatives than third cousins. Additional results on full sibships, half sibships and first cousinships (not shown) have all confirmed the findings we have discussed. In particular, asymptotic bias in heritability increases with remoteness of relationship when the additive genetic correlation matrix is mis-specified.

Formulas in **METHODS** do not associate $G_{t}$ or $G_{f}$ with specific kinship measures. They are applicable to all choices of genetic correlation matrices. The decision to assume realized *vs.* estimated kinship as the appropriate kinship measure for $G_{t}$ depends on one’s belief on whether genetic correlation is best captured by IBD sharing or by IBS matching at genotyped markers. Taking the IBS perspective, Jiang *et al.* (2016) provided a theoretical justification for consistency of heritability estimates in genome-wide association studies (GWAS) under mis-specified linear mixed model. The authors assumed that $G_{t}$ takes the form of the classic GRM, but the fitted model is mis-specified in the sense that only a subset of the GWAS SNPs are actually causal. This setup falls within the more general discussions in this paper. We have shown that fitting a covariance matrix that is broader than truth causes little asymptotic bias (*e.g.*, Figure 3B).

A model with only two random effects is attractive for ease of computation and interpretability. In particular, an eigen-transformation is possible so that variance covariance matrix of the transformed trait is diagonal, and it depends on the appropriate genetic correlation matrix only through the eigenvalues. This simplification is not possible with more than two random effects. The asymptotic properties of heritability estimates under more complicated random effects models will be a topic of future investigation.
